# Assessment of the bone quality of black male athletes using calcaneal ultrasound: a cross-sectional study

**DOI:** 10.1186/1743-7075-5-13

**Published:** 2008-05-20

**Authors:** Emmanuel P Laabes, Dorothy J VanderJagt, Michael O Obadofin, Ayuba J Sendeht, Robert H Glew

**Affiliations:** 1 Department of Family Medicine, Jos University Teaching Hospital, PMB 2076, Jos, Nigeria; 2 Department of Biochemistry and Molecular Biology, University of New Mexico, Albuquerque, New Mexico, USA; 3 Research Laboratory, Department of Obstetrics and Gynaecology, Faculty of Medical Sciences, University of Jos, Nigeria

## Abstract

**Background:**

Lifestyle, genetics and environmental factors are established determinants of bone density. We aimed to describe the bone characteristics of competitive top-ranked Nigerian male athletes using calcaneal ultrasound and to assess whether intensive training promotes higher bone density in an environment with reportedly low calcium intake; to compare the bone characteristics of footballers with runners and other sportsmen; and to assess the correlation of stiffness index (SI) with activity level, since energy expenditure correlates with length of training and by extension, magnitude of skeletal loading.

**Methods:**

We recruited 102 male athletes: these included football (n = 68), running (n = 15), handball (n = 7), taekwando (n = 6), cycling (n = 2), judo (1), badminton (1) and high jump (1). Anthropometric data were first recorded on a structured form and energy expenditure was indirectly estimated with a validated questionnaire. Bone density was assessed using the Lunar Achilles+ calcaneal ultrasonometer.

**Results:**

The mean age of athletes was 25 ± 6 years. The means of BMI and energy expenditure were 21.9 ± 2.0 kg/m^2 ^and 35.0 ± 13.7 kcal/kg/day, respectively. Footballers were younger (p < 0.001) and heavier (p < 0.001) than runners. Football was a significant determinant of BUA independent of age, BMI and energy expenditure (p = 0.001). Football was also a significant determinant of SOS independent of age, height, weight and BMI (p < 0.001). The mean SI was 127 ± 16 and the median T-score was 0.82 (-1.88, 3.35). The mean SI of footballers (130 ± 15), runners (130 ± 12) and other sportsmen (115 ± 18) differed significantly (p = 0.001). Multivariate analyses revealed that football (p < 0.001) and running (p < 0.001) were significant determinants of SI independent of age and BMI. Footballers when compared with other sportsmen had a higher mean SI independent of age and BMI (p < 0.001). Age was not correlated with SI. The median T-score of footballers, 0.94 (-1.0, 3.35) was higher than that of other sportsmen.

**Conclusion:**

Repetitive skeletal loading at the heel has the potential to improve bone density in black male athletes. The magnitude of increase may be higher in medium impact sports such as soccer and running compared with low or non-impact sports such as judo or taekwando, and is independent of age and BMI. However, future longitudinal data will be required to support our observations.

## Background

Bone health is an important public health issue worldwide. The World Health Organization defines osteoporosis as bone mineral density (BMD) >2.5 standard deviations below the mean for a normal young adult [1]. Osteoporosis has been reported to occur in 20% of men in the U.S. [2]. In the U.K., direct medical costs to the National Health Service attributed to osteoporosis have been estimated at £411 million per year [3]. There is a paucity of reports from sub-Saharan Africa regarding bone quality and osteoporosis.

Despite the well established beneficial effect of consistent sporting activity on indices of bone health, the risk of sport-related bone injury is real and can not be discounted [4-6]. Skeletal injuries are relatively uncommon in sportsmen, but their occurrence may have devastating health and career implications[7]. The factors related to fracture risk include genetics, lifestyle and nutrition[8,9]. Both a high physical activity level and adequate calcium intake have been reported to be associated with higher BMD in male athletes [10]. However, animal studies have shown that exercise-induced changes in the bone of growing mice were gender-specific [11].

Peak bone mass, achieved by early adulthood, depends on calcium intake during childhood and adolescence [12-14]. The diets of populations in Nigeria and other areas of sub-Saharan Africa have been reported to be low in calcium and are based on cereal staples that contain chelators such as phytates [15,16]. In a recent dietary study conducted in northern Nigeria, the mean dietary calcium intake of adult males was 551 mg/day, a figure that is below the U.S. recommended dietary intake of 1000 mg/day [17]. In a study of rachitic children in Nigeria, we reported that the median intake of dietary calcium was 191 mg/day and was similar for rachitic children and controls [15].

The present study was aimed at assessing the characteristics of the bones of high-performance, top-level competitive professional athletes in Nigeria, using calcaneal ultrasound. The purpose of the study was three-fold: first, to describe the bone characteristics of competitive Nigerian professional athletes and assess whether sporting activity promotes higher bone density; second, to compare the bone characteristics of male football (soccer) players, runners and athletes training for other sports. An ancillary aim was to assess the correlation of stiffness index (SI) with activity level.

Dual-energy X-ray absorptiometry (DXA) is widely accepted as the gold standard for assessment of BMD – a surrogate for bone strength [18]. Although bone density measured by DXA shows a high correlation with bone strength, about 25–30% of the variation in bone strength results from the combined effects of microstructure, architecture and remodeling [19,20]. Bone densitometry techniques are therefore incapable of measuring the direct effect of these factors on bone strength. Since acoustic energy in the form of ultrasound interacts with bone tissue in a different way from electromagnetic ionizing radiation, it has the potential to provide additional information on the biomechanical properties of bone which can not be obtained using bone densitometry [21]. For example, sound can be modified by the bone's mass, structure and composition, to provide additional information related to the mechanical strength of bone. This information is provided as bone stiffness index (SI); a composite clinical measure derived from both the broadband ultrasound attenuation (BUA) and speed of sound (SOS) measurements [22]. Bone quality assessed as SI has been shown to correlate with hip or spine BMD, and to be as useful for predicting fracture risk as DXA [23-25]. Furthermore, calcaneal ultrasound has been shown to be able to detect variations in bone SI based on activity level [26]. In addition, ultrasound is relatively inexpensive, simple to conduct and involves no ionizing radiation.

We therefore recruited professional male athletes involved in a variety of sports at the Rwang Pam township stadium in Jos, Nigeria, and measured their bone characteristics using calcaneal ultrasound. We also estimated average daily energy expenditure with a validated questionnaire. This report documents the SI, BUA, SOS and T-scores of active Nigerian sportsmen categorized by sporting activity.

## Methods

### Study area

Jos is located in north-central Nigeria and lies between latitude 09°52'N and longitude 08°54'E at an elevation of 1285 m above sea level[27]. Rwang Pam township stadium, located in the heart of Jos metropolis, is a 15,000 capacity football stadium with facilities for track-and-field as well as in-door sports. Jos University Teaching Hospital (JUTH) is a federal tertiary care facility located about 1.5 kilometers from the stadium.

### Study population

All professional male athletes aged 18–49 years, presenting for early morning training in various sports within the stadium were eligible for recruitment into the study. All athletes had undergone at least three months of intensive training before the commencement of the study. The study period coincided with training toward national camping for selection of athletes to the All-Africa games held July 11^th ^to 23^rd^, 2007, in Algiers. Subjects with a reported diagnosis of cancer, liver disease, chronic kidney disease, soft tissue injury, or a bone fracture precluding training within the preceding 12 weeks were excluded. Equally, reported past or current intake of steroids (prescribed or over-the-counter) was a basis for exclusion. Overall, 68 football players, 15 runners (sprinters, middle distance runners and marathoners), 7 handball players, 6 taekwando players, 2 cyclists, and 1 each of judo, badminton and high jump players met the inclusion criteria. All recruited footballers played for first division teams in the Nigerian professional league. The disproportionate composition of athletes was due to the paucity of sportsmen in certain categories. The study was approved by the Ethical Committee of JUTH, the Human Research Review Committee of the University of New Mexico, and the Plateau State Directorate of Sports. Written informed consent was obtained from all subjects.

### Study protocol

This study was carried out in May, 2007. The coaches assisted with recruitment. The purpose of the study including any risks and benefits were explained to all subjects. Consecutive, eligible, consenting sportsmen were then transported to JUTH where anthropometric and calcaneal ultrasound measurements were conducted. In addition, a validated questionnaire was used to obtain information on each athlete's training routine, as exemplified by training within the preceding two weeks.

### Anthropometric assessment

Height was measured without shoes and headgear, using a portable stadiometer calibrated to the nearest 0.1 cm. Weight was measured with subjects wearing light clothing and without shoes, to the nearest 0.1 kg, using a scale (Metro, 2001 Model, USA, sensitivity, 0.5 kg) calibrated with known weight daily. The body mass index (BMI) was defined as the quotient of the weight in kilograms and the square of the height in meters, expressed as kg/m^2^

### Calcaneal ultrasound

Ultrasound measurements of the calcaneus were obtained using the Achilles+ (Lunar Corporation, Madison, WI, USA) according to the manufacturer's instructions. Each subject was seated with his right foot resting in the heel bath of the instrument. The ultrasound instrument then automatically introduces surfactant-containing warm water into the heel bath, and takes measurements of the BUA and SOS transmission. This ultrasound technique has been fully described elsewhere [28]. BUA is defined as the slope of the regression line derived from the ratio of the signal amplitude of the calcaneus to that of water (reference) at each frequency of ultrasound (dB/MHz). SOS refers to the speed of the sound wave traveling through the calcaneus, measured in m/s. The stiffness index was calculated for each subject using the equation SI = (0.67 × BUA) + (0.28 × SOS) - 420 [22]. The SI is a single clinical measure whose calculation has a 50% contribution from BUA and a 50% contribution from SOS; it has a lower precision error than either BUA or SOS alone. Calibration of the instrument was monitored using a phantom heel provided by the manufacturer. Reproducibility was validated by running a daily quality assurance check before the onset of measurements. The BUA and SOS measurements had good precision (coefficient of variation, 9% and 2% respectively).

Although a recent population-based observational study of sedentary subjects reported significant differences in calcaneal BMD between the dominant and non-dominant foot, there is currently no consensus as to which foot should be measured for the diagnosis of osteoporosis or prediction of fracture risk [29]. We compared left versus right foot measurements in a handful of our subjects and did not demonstrate any difference in SI between the left and right foot. We therefore conducted SI measurements on the right foot, which was the dominant foot in most athletes.

The T-score was calculated as follows: T-score = (subject SI - mean SI of the reference group)/(standard deviation of the reference group). The reported mean SI of 115 ± 17 for healthy Nigerian males aged 20–29 years was used as the mean SI of the reference group [28].

### Activity questionnaire

Each subject was administered a validated physical activity questionnaire developed by the US National Health Interview Survey [30,31]. Although the questionnaire assessed the physical activity of each subject for the preceding two weeks, we sought to establish each athlete's training routine beginning from the onset of a professional career. This assessment allowed for a calculation of the average daily energy expenditure for each athlete, which was taken as the athlete's habitual training intensity in the preceding three months. Using a standardized scale, self-identified individual activities were classified into metabolic equivalents (METs) which were then converted to energy expenditure values expressed as kilocalories per kilogram per day [32]. Energy expenditure for a particular activity was computed by multiplying the weekly frequency of activity by its duration in hours and its METs code derived from a standard table {i.e. frequency (times/wk) × duration (in hrs) × METs}. The daily energy expenditure in kcal/kg/day was then derived by dividing the weekly energy expenditure by seven. The calculation was done for each reported activity and the total energy expenditure in kcal/kg/day was taken as the sum of the daily energy expenditures for all activities.

### Statistical Methods

Data were analyzed with EpiInfo 3.4 (CDC, Atlanta Georgia). Results are expressed as mean and standard deviation for continuous normally distributed variables. Linear regression was used to assess correlations between continuous variables. Multivariate analyses were used to establish the independent determinants of BUA, SOS and SI. Multivariate analysis was used to assess confounding by age and BMI, of the observed difference in SI between footballers and other sportsmen. The t-test and one-way ANOVA were used to compare group means where appropriate. Bartlett's test for homogeneity of variances was used to assess suitability of data for ANOVA. Owing to multiplicity of comparisons, we used p < 0.01 to indicate statistical significance based on the Bonferroni correction.

## Results

### Demographic characteristics of subjects

A total of 106 male athletes gave consent for recruitment, 4(3.8%) were excluded because they experienced an injury which precluded training in the preceding 12 weeks. Thus 102 athletes concluded the study and their data were analyzed. The mean age of recruited athletes was 25 ± 6 years (range, 17–47) (Table 1). The mean age of footballers (23 ± 4 years), runners (31 ± 8 years), and other sportsmen (27 ± 6 years) differed significantly (p < 0.001). Footballers were younger than runners (p < 0.001). Other sportsmen when combined were also younger than runners (p = 0.005, Table 2). The majority of the subjects, 70(68.6%) were in the 20 to 29 year age group. Overall, there were 68 (66.7%) footballers, 15(14.7%) runners, 7(6.8%) handball players, 6 (5.9%) taekwando players and 2(1.9%) cyclists. Judo, wrestling, badminton and high jump all had one (0.9%) player each.

**Table 1:** Anthropometric characteristics, activity level and ultrasound parameters of 102 Nigerian sportsmen

**Characteristic**	**Mean ± SD**	**Median (range)**
Age (years)	25.0 ± 6.00	24.0 (17, 47)
Weight (kg)	65.5 ± 8.00	65.0 (46.2, 85.2)
Height (m)	1.73 ± 0.64	1.72 (1.57, 1.89)
BMI (kg/m^2^)	21.9 ± 2.00	21.8 (17.8, 29.6)
Activity (kcal/kg/day)	35.0 ± 13.7	35.0 (4.00, 65.5)
BUA (dB/MHz)	141 ± 11	142 (111, 161)
SOS (m/s)	1618 ± 40	1617 (1534, 1778)
SI	127 ± 16	129 (83, 172)
T-Score	0.69 ± 0.95	0.82 (-1.88, 3.35)

BMI = body mass index.

BUA = broadband ultrasound attenuation.

SOS = speed of sound.

SI = stiffness index.

**Table 2:** Comparison of footballers, runners and other sportsmen by Means of anthropometric, activity and ultrasound parameters

**Characteristic**	**Footballer^a ^(n = 68)**	**Runner^b ^(n = 14)**	**Other^c ^(n = 19)**	**P-value**			
				
				a*b*c	a*b	a*c	b*c
Age (years)	23 ± 4	31 ± 8	27 ± 6	<0.001	<0.001	0.005	0.06
Weight (kg)	66.3 ± 7.0	58.7 ± 6.0	67.6 ± 10.3	0.001	<0.001	0.52	0.005
Height (m)	1.73 ± 0.6	1.69 ± 0.6	1.76 ± 0.7	0.005	0.01	0.09	0.004
BMI (kg/m^2^)	22.1 ± 1.8	20.6 ± 1.9	21.7 ± 2.4	0.02	0.004	0.36	0.17
Activity (kcal/kg/d)	35 ± 13	27 ± 12	40 ± 16	0.02	0.02	0.20	0.01
BUA (dB/MHz)	142 ± 9	143 ± 11	135 ± 13	0.02	0.62	0.01	0.06
SOS (m/s)	1625 ± 40	1623 ± 28	1590 ± 39	0.003	0.84	0.001	0.01
SI	130 ± 15	130 ± 12	115 ± 18	0.001	0.96	<0.001	0.01
T-score	+0.85 ± 0.88	+0.86 ± 0.69	-0.01 ± 1.0	0.001	0.96	0.001	0.01

P < 0.01 indicates statistical significance (Bonferroni correction).

a*b*c = comparison of footballers (a) with runners (b) and other sportsmen (c).

a*b = comparison of footballers (a) with runners (b).

a*c = comparison of footballers (a) with other sportsmen (c).

b*c = comparison of runners (b) with other sportsmen (c).

Others include handball, high jump, taekwando, wrestling, cycling, badminton and judo.

### Anthropometry

Table 1 summarizes the anthropometric characteristics of the sportsmen. The mean weight of the athletes was 65.5 ± 8.0 kg, the mean height was 1.73 ± 0.64 m and the mean BMI was 21.9 ± 2.0 kg/m^2^. Comparisons of footballers, runners and other sporting categories in terms of anthropometry are shown in Table 2. Footballers (66.3 ± 7.0 kg) were heavier than runners (58.7 ± 6.0 kg), p < 0.001. Other sportsmen (67.6 ± 10.3 kg) were also heavier than runners, p = 0.005. There was no significant difference in BMI between footballers (22.1 ± 1.8 kg/m^2^), runners (20.6 ± 1.9 kg/m^2^) and other sportsmen (21.7 ± 2.4 kg/m^2^), p = 0.02. However, when compared with runners, the mean BMI of footballers was significantly higher, p = 0.004.

### Physical activity measurements

The mean energy expenditure was 35.0 ± 13.7 kcal/kg/day (Table 1). One-way analysis of variance with Bonferroni correction did not reveal any significant difference in the mean activity levels of the sportsmen, p = 0.02 (Table 2).

### Ultrasound parameters

The mean BUA was 141 ± 11 dB/MHz and the mean SOS was 1618 ± 40 m/s. The mean SI of the sportsmen was 127 ± 16 and the median T-score was 0.82 (-1.88, 3.35). The ultrasound parameters of the sportsmen are summarized in Table 1. The age group 20 to 29 years had the highest mean SI of 129 ± 16.

Table 2 shows a comparison of the athletes in terms of ultrasound parameters. The mean BUA of the three groups did not differ significantly, p = 0.02. However, their mean SOS differed significantly, p = 0.003. The mean SOS of footballers (1625 ± 40 m/s) was significantly higher than that of other sportsmen (1590 ± 39 m/s), p = 0.001. Football was a significant determinant of BUA independent of age, BMI and energy expenditure (p = 0.001). Equally, football was a significant determinant of SOS independent of age, height, weight and BMI (p < 0.001). There was a significant difference in the mean SI of footballers (130 ± 15), runners (130 ± 12) and other sportsmen (115 ± 18), p = 0.001. Multivariate linear regression analysis revealed that football (p < 0.001) and running (p < 0.001) were significant determinants of SI independent of age and BMI in this cohort. There was no significant difference in the mean SI of footballers compared with runners, but the mean SI of footballers was higher than that of other sportsmen (p < 0.001). The difference in mean SI between footballers and other sportsmen remained significant after controlling for age and BMI in the multivariate model (p < 0.001). In terms of T-score, there was a significant difference in median T-scores between the three groups; p = 0.001. The median T-score of footballers, 0.94 (-1.0, 3.35) was significantly higher than that of other sportsmen 0.0 (-1.88, 1.88)), p = 0.001, but not significantly different from that of runners, 0.88 (-0.59, 2.35), p 0.96.

The highest mean SI of 130 was recorded in both footballers and runners, while cyclists had the lowest mean SI of 100. Similarly, footballers and runners recorded the highest median T-scores compared with the rest of sportsmen. Table 3 shows the means of SI and median T-scores for the individual sports.

**Table 3:** Mean stiffness index and median T-scores of subjects categorized by sports

**Sport**	**Mean Stiffness Index (± SD)**	**T-Score Median (range)**
Football (n = 68)	130 (± 15)	0.94 (-1.0, 3.35)
Running (n = 15)	130 (± 12)	0.88 (-0.59, 2.35)
Others* (n = 19)	115 (± 18)	0.0 (-1.88, 1.88)
Handball (n = 7)	120 (± 16)	0.47 (-1.12, 1.71)
Taekwando (n = 6)	117 (± 18)	0.27 (-1.88, 1.0)
Cycling (n = 2)	100 (± 16)	-0.89 (-1.53, -2.04)

Others* refer to the combination of all remaining categories besides football and running. Wrestling, judo, high jump and badminton had only one subject each.

### Correlation of stiffness index with age, anthropometry and activity

There was no significant correlation of SI with any of the following parameters: age, weight, BMI, and energy expenditure. The T-score calculations showed high precision (T-score perfectly correlated with SI, r = 1.0, p < 0.001). We were unable to control for other possible confounders of the effect of sporting category on ultrasound indices of bone strength, like calcium intake, ethnicity and years of training, since we did not collect such data.

## Discussion

Our study had three aims, the first being to describe the bone characteristics of competitive, high-performance Nigerian male athletes and to assess if sporting activity promotes higher bone density. Secondly, we aimed to determine if there were any significant differences in SI between different kinds of sports. A third aim was to assess relationships between SI and training intensity as reflected in the average daily energy expenditure of the athletes.

The main finding of our study was that athletic training promotes higher bone density in black male athletes by about one T-score unit. This statement was true in the case of football and running (sprinters, middle-distance runners and marathoners), but not high jump, cycling, karate, handball, badminton and Judo.

In a recent attempt to describe the ultrasound characteristics of 756 healthy adult Nigerians, VanderJagt and associates reported values of 137 dB/MHz, 1580 m/s and 115 for BUA, SOS and SI respectively, in males aged 20–29 years [28]. We report correspondingly higher values for all three ultrasound parameters in our subjects of the same age decade. The high bone ultrasound characteristics of the athletes may be attributed to intensive physical training or be entirely unrelated to it. Other yet unidentified factors such as optimal calcium intake may account for our findings.

The magnitude of the impact of physical training on SI observed in our study is comparable to that reported in other studies. For example, using peripheral DXA, Ruffing and colleagues reported an increase in mean calcaneal BMD above one standard deviation in a cohort of 755 physically active male US military cadets [10]. Ruffing and co-workers observed a significantly higher BMD at the heel, hip and spine in African-Americans compared with their Asian and Caucasian counterparts. Prior exercise level was positively related to BMD regardless of race. Similarly, Wittich and co-workers, using DXA, reported a 12.3% increment in BMD of professional first division male football (soccer) players compared with age- and BMI-matched controls [33]. The increase in BMD did not differ between younger players (< 7 years of practice) and older players (> 7 years of practice). The increase in BMD of footballers was greater in the pelvis and legs compared with the arms and trunk. Dairy calcium intake was similar in footballers and controls and was unrelated to BMD. All the footballers were in the second or third decade of life, similar to our recruited soccer players. The paucity of this type of data from sub-Saharan Africa precluded comparison of our findings with those of other African countries.

The literature is replete with reports demonstrating a correlation of athletes' training volume and duration with bone strength [34-36]. The calcaneus contains 75–90% cancellous bone by volume, it is eight times metabolically more active than compact bone and readily shows age and activity related changes [37]. We did not observe any correlation of SI with activity level. Gutin and colleagues admitted the difficulty associated with accurate measurement of physical activity level, which tends to reduce the chances of finding a significant correlation of bone quality with physical activity [38]. Since bony tissue reacts slowly to habitual loading, it is more appropriate to seek correlations of SI with long term training variables, in particular, a combination of long term training intensity and duration, as suggested by Walker and Holm [39]. However, while it is easy to assess training duration, the same can not be said of training intensity or volume over a long period, for obvious reasons of recall bias. Messenger and colleagues admitted rejecting interviewee responses to questions pertaining to long term training variables owing to unreliability [26]. Furthermore, infrequent bursts of intensive training over many years may not confer any advantage in terms of SI compared with consistent training. Therefore, we used information on training history over the preceding two weeks to estimate the athletes' habitual average daily energy expenditure in the long term. This training history represented the athlete's habitual training over the preceding 3 months. In the absence of information on long term training variables, assessing a correlation of SI with energy expenditure over three months may not be as informative. However, If the energy expenditure estimates for all athletes were accurate and represented long term training intensity, our failure to find a relationship between SI and activity would suggest that the qualitative aspects of a particular kind of training are important determinants of SI. This assertion is supported by our failure to find a significant difference in the mean SI values of footballers and runners. For example, football and running may recruit muscle groups that ultimately exert a positive effect on bone metabolism. Wittich and co-workers previously reported a positive correlation of BMD with muscle mass independent of height, weight and lean body mass in professional male soccer players [33]. In this regard, it would be interesting to study biochemical markers of bone metabolism in Nigerian athletes participating in different kinds of sports. Furthermore, as reported by Wiswell and colleagues, a lack of correlation between SI and energy expenditure may be explained by the fact that consistent training for long periods by these professional athletes might have led to a situation where further increases in energy expenditure are not accompanied by increases in SI [40].

Previous reports have demonstrated that sports stressing axial loads on the skeleton, such as weight lifting, lead to significant increases in BMD in the lumbar spine [41]. The same effect has been demonstrated on calcaneal BMD [42]. It has also been reported that varying degrees of impact forces on the calcaneus exert varying effects on its BMD [43]. It is generally agreed that the magnitude of skeletal loading is less in soccer and running when compared with professional weight-lifting; with the magnitude of skeletal loading being higher in football and running when compared with swimming and cycling [44]. In agreement with Nikander and colleagues, we found the highest mean SI in the soccer players and runners compared with other sportsmen, which also concurs with previous reports from Germany and Finland [41-43]. This difference in SI persisted after controlling for age and BMI, p < 0.001. We also observed similar differences in terms of SOS and T-scores between the three categories-footballers, runners and other sportsmen. Although footballers were younger than runners, the mean SI was similar in the two groups, suggesting that activity level may have a more substantial contribution to SI than age in this cohort.

Age and training volume are known to confound indices of bone strength; the younger age of the footballers did not confer a disadvantage in terms of SI. Instead, the footballers had higher mean SI despite the fact that some of them were yet to achieve maximum bone strength. The lack of a correlation of age with SI further suggests that age did not confound the SI difference between footballers and other sportsmen. Equally, the lack of a significant difference in the bone indices of footballers and runners may be due to our small sample size as reflected in the wide confidence intervals for our estimates. Furthermore, since most male children are exposed to soccer right from kindergarten, our demonstration of a significant difference in SI between footballers and other sportsmen suggests that exposure to amateur soccer early in life may not confer any advantage on SI of sportsmen. Rather, consistent physical training in a professional capacity may account for the observed differences. Most of our non-soccer athletes confessed to regularly playing soccer for fitness training or leisure.

Our finding that the runners were the lightest of the athletes in the three sports categories support the observation that runners have a tendency to regulate their weight in order to remain competitive. However, this observation may be confounded by our inability to recruit more runners.

In terms of significance, the results of our study demonstrate that both the intensity and nature of sporting activity are important determinants of SI in black athletes.

Our data imply that, controlling for age and BMI, a reduced fracture risk may be associated with football and running compared with say judo or badminton in this cohort.

The study had several limitations: in view of the well established relationship of calcium intake with bone quality and lifetime fracture risk [45], dietary information would have provided an estimate of calcium intake by the athletes; and would have allowed an assessment of a correlation of SI with calcium intake. However, we have no reason to believe that the calcium intake of the athletes would differ from the general population, since the athletes' diets were similar to those of other household members. Furthermore, our interaction with the athletes revealed a lack of access to professional nutritional advice. Biochemical analyses of quantitative markers of bone metabolism would have allowed for a comparison of different sports with activity levels and quantitative markers of bone turnover, such as N-telopeptides (NTx) and alkaline phosphatase. In light of the well known limitations of BMI, it would have been helpful to obtain information regarding body composition. For example, two different individuals with the same BMI may have different proportions of body fat and fat-free mass. We were unable to control for the influences of socio-economic status, ethnicity and years of training on the relationship of sports with SI because we did not collect such data. Furthermore, the study would have benefited from the availability of more athletes in the categories other than football and running.

Future studies that are planned include measuring biochemical markers of bone turnover (e.g., NTx, bone-specific alkaline phosphatase) in footballers and runners versus athletes in other sports such as cycling, handball and taekwando. In addition, we plan to estimate body composition via bioelectrical impedance analysis [46,47].

## Conclusion

In conclusion, medium impact sports positively influence bone density in black male athletes compared with low or non-impact sports. In particular, football and running have the potential to increase calcaneal SI in black male athletes by one T-score unit compared with judo or taekwando. Our data suggest that both the intensity and nature of sporting activity are important determinants of SI in black athletes. These observations if supported by future longitudinal data will prove useful in the prediction of fracture risk in black athletes. However, our small sample size resulting in the wide confidence intervals for our estimates limit the conclusions that can be drawn.

## Competing interests

The authors declare that they have no competing interests.

## Authors' contributions

Both EPL & RHG conceived and designed the study, recruited subjects, performed ultrasound measurements, analyzed and interpreted data, drafted and edited the manuscript. DJV took part in conception and design of the study and critical editing of the manuscript. MOO took part in data collection, analysis and interpretation and the writing of the manuscript. AJS recruited subjects, performed ultrasound measurements and took part in data analysis and interpretation. All authors read and approved the manuscript.
